# Is dorsal inlay graft (DIG) with TIP repair superior to TIP alone for primary hypospadias? A randomized clinical trial

**DOI:** 10.1186/s12887-025-06247-7

**Published:** 2026-01-26

**Authors:** Mohamed Ahmed Negm, Ahmed Abdelmoneim Abdelrasheed, Mohammad Daboos, Mohammed M. Khedre, Mohamed F. Abdelrahman, Nezar Abd Elrouf Abo-Halawa, Ibrahim Ali Ibrahim, Mohamed Ali Shehata

**Affiliations:** 1 Pediatric Surgery Unit, Qena Faculty of Medicine, Qena University Hospitals, South Valley University, Qena, 83523 Egypt; 2 Pediatric Surgery Department, Al-Azhar University, Cairo, Egypt; 3 Pediatric Surgery Unit, Faculty of Medicine, Minia University Hospitals, Minia University, Minia, Egypt; 4 Pediatric Surgery Department, Faculty of Medicine, Assiut University Hospitals, Assiut University, Assiut, Egypt; 5 Pediatric Surgery Unit, Faculty of Medicine, Tanta University Hospitals, Tanta University, Tanta, Egypt

**Keywords:** Hypospadias, TIP, Snodgraft, Urethral plate (UP), Dorsal inlay graft (DIG)

## Abstract

**Background:**

Tubularized incised plate urethroplasty (TIP) is a widely practiced technique for hypospadias repair; however, it remains associated with complications, particularly meatal and/or neourethral stenosis. Grafting the incised plate with a dorsal inlay graft (DIG) during TIP has been proposed as a preferred modification to address these complications. Although several studies have investigated DIG in cases with a narrow urethral plate (UP), no current consensus exists regarding its use as an adjunct to TIP repair in different types of UP.

**Objectives:**

The current study aimed to investigate whether DIG with TIP repair is superior to TIP in different types of UPs regarding surgical and cosmetic outcomes.

**Methods:**

In a comparative, randomized study conducted from January 2023 to December 2024, patients with primary hypospadias without chordee were randomly assigned to two groups: Group 1 underwent repair using TIP, and Group 2 underwent repair using the DIG with TIP technique. Short- and mid-term outcomes were compared between the two groups.

**Results:**

During the study period, 579 primary hypospadias cases met the inclusion criteria (290 in Group 1 and 289 in Group 2). The median age in both groups was 22 months (IQR, 13–36.25 months). Operative time was significantly longer in Group 2 (*P* = 0.001). Among patients with a narrow UP, surgical outcomes were better in Group 2, with statistically significant improvement in neourethral and meatal function (*P* = 0.001). In Group 1, meatal stenosis developed in 25 patients (8.62%); 14 cases presented early and improved with regular dilation, whereas 11 cases presented after 6 months and required meatoplasty. In patients with a wide UP (> 8 mm), better results were observed in Group 2, although the difference was not statistically significant. Regarding the final position of the meatus at the glans tip, significance was noted in Group 2 (*P* = 0.008). The follow-up period ranged from 6 to 23 months (mean ± SD: 13.76 ± 5.68). The hypospadias objective scoring evaluation (HOSE) score at the 6th month demonstrated statistical significance in Group 2.

**Conclusions:**

Adding a DIG to TIP repair potentially reduces the risk of neourethral and meatal complications in patients with a narrow UP (4–8 mm). However, in wide UP (> 8 mm), comparable outcomes were observed in both groups. The dorsal inlay graft with tubularized incised plate (DIGTIP) is associated with longer operative times and greater technical demands; therefore, it should be applied only in selected patients and according to the surgeon's preference.

**Trial registration:**

[NCT07086963] "Retrospectively registered" Date of registration [Jul 17 2025] https://clinicaltrials.gov/study/NCT07086963 and Unique identifying number [SVU/MED/SUR011/4/22/12/519].

## Introduction

Tubularized incised plate urethroplasty (TIP) is one of the most widely used techniques globally because of its versatility in repairing various types of hypospadias [1, 2]. Nevertheless, meatal stenosis, neourethral stricture, and urethrocutaneous fistula (UCF) remain the most common complications [3–5]. TIP repair primarily depends on the quality of the urethral plate (UP) and glanular configuration; therefore, these complications are associated with anatomical factors, particularly the width of the UP and the size of the glans, as well as technical factors and the surgeon's experience [6–8].

According to Snodgrass, widening the UP through a deep incision can overcome a narrow plate, based on the hypothesis that the incised plate will become epithelialized [1, 9, 10]. However, there is no universal agreement regarding the healing of the raw area of the incised plate, as retraction and fibrosis may lead to narrowing of the neourethra [3, 4, 10]. The dorsal inlay graft (DIG) is a vital modification adjunct to TIP repair, as it potentially prevents or minimizes stricture formation in the neourethra and/or urethral meatus. It was first described by Kolon and Gonzales in 2000 [11].

Grafting the incised UP has been reported by several authors as a means to improve operative outcomes, particularly the function of the neourethra and urethral meatus [12–16]. Current studies have compared standard TIP with grafted TIP (GTIP) in cases of poorly developed or narrow UP, with only a few studies addressing patients with a UP wider than 8 mm [15, 16]. Nevertheless, complications are still reported in some cases with a wide UP. The authors believe that width and depth are not the only determinants of UP characteristics; the extension of UP to the glans tip is also important. However, these parameters remain a subjective assessment [13–17]. Moreover, to the best of the researchers' knowledge, no study has compared the outcomes of both techniques across different types of UP.

This study aimed to investigate whether DIG with TIP repair is superior to TIP alone in various kinds of UPs with respect to surgical and cosmetic outcomes.

## Patients and methods

### Study design

This prospective, randomized study included all patients who presented with primary hypospadias without chordee during the study period from January 2023 to December 2024.

All patients who met the study criteria were enrolled according to the following parameters:

Inclusion criteria:

- Primary hypospadias candidates for one-stage repair
- Uncircumcised cases
- Pediatric age group
- Patients with regular follow-up

Exclusion criteria:

- Presence of chordee or urethral anomalies requiring division of the UP or staged repair
- Small glans < 12 mm and/or UP < 4 mm
- Glanular hypospadias
- Previous testosterone supplementation for small glans or other reasons

### Method of randomization and sample size calculation

Patients were randomized into two groups using sealed envelopes labeled as "Group 1" or "Group 2," selected by the operating theater chief nurse. An independent statistician approved the sample size.

### Ethics statement

Written informed consent was obtained from the parents of all patients before enrollment in this study.

### Patient assessment

For enrolled patients, the following parameters were documented: glanular size, UP width, and whether the UP extended from the hypospadias opening to the glans tip.

### Patient allocation and enrollment

Patients who fulfilled the inclusion criteria were enrolled and randomly allocated into one of two groups:

- Group 1: Repaired with standard TIP
- Group 2: Repaired with TIP with DIG from the inner prepuce

### Surgical steps

**Fig. 1:**
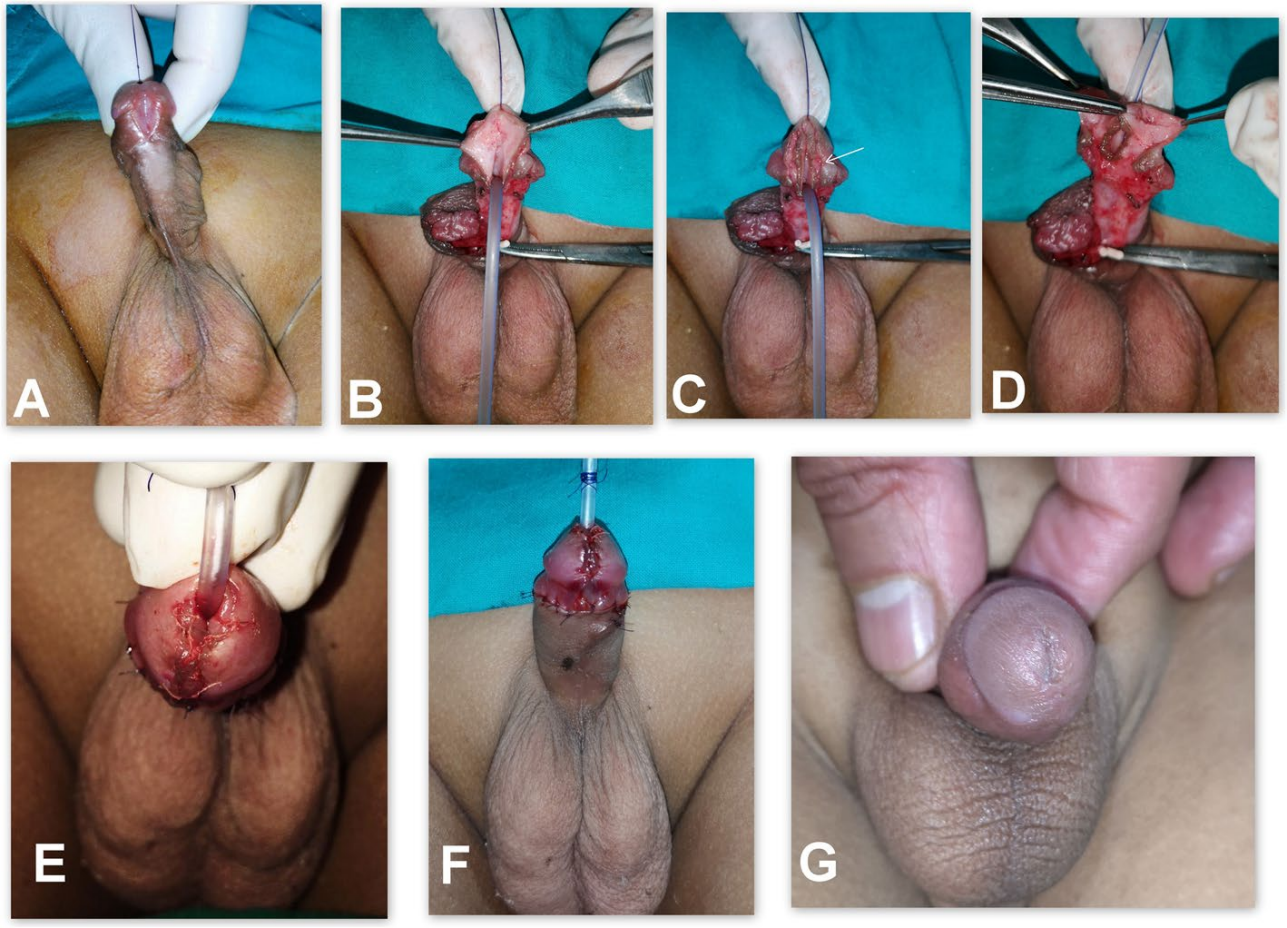
Surgical steps of TIP repair. **A** Patient with distal penile hypospadias. **B** Deep midline incision of the urethral plate with proximal extension beyond the hypospadic meatus to avoid stricture commonly occurred in this area and prevent narrowing of urethral lumen during urethroplasty and spongioplasty. **C** Creation of urethral edges and glanular wings, creation of good mucosal collar, maintaining its continuity with proximal glans at point C (white arrow), degloving with preservation of well vascularized skin and avoid compression of skin by tourniquet. **D** Wide glanular wings dissection, starting urethroplasty at mid glans to avoid meatal stenosis (as recommended by Snodgrass). **E**, **F** The end of the procedure with maintaining adequate meatus around the catheter, good mucosal collar and vascularized skin coverage without midline closure. **G** The same patient during follow up

**Fig. 2:**
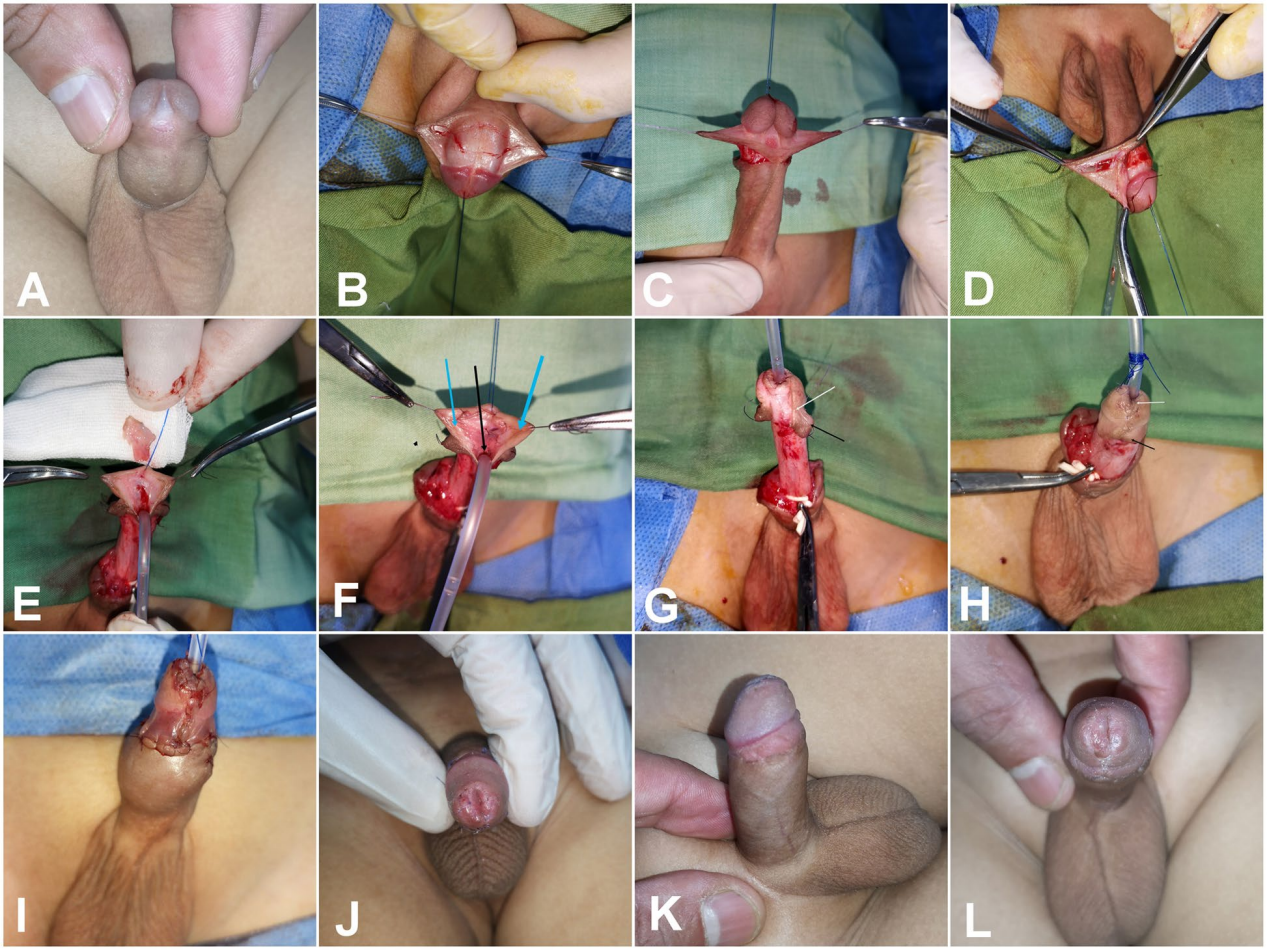
Demonstrating detailed surgical steps of DIGTIP**. A** Patient with sub coronal hypospadias with wide and shallow UP. **B**, **C** Circumcising incision was designed to achieve about one cm mucosal collar and outline the donor area for free inner face preputial mucosal graft. **D** Preparation donor area for inner face preputial mucosal free graft. **E** Midline incision of UP extended proximally beyond the hypospadic meatus to avoid stricture in this area and adjusting the graft size to the raw area of the incised plate. **F** Graft fixation (using 7/0 polyglactin sutures) to the edges of the urethral plate (arrows) with a tiny incisions over the graft. **G** Glanular wings dissection with preserving a good length of viable mucosa (about 1 cm, black arrow) and maintaining the continuity between the proximal glans and the ventral end of the mucosal collar at point C (white arrow).Urethroplasty using 7/0 Vicryl starting at the point of bifurcation of spongiosal tissue (proximal to the hypospadias meatus), including the two spongiosum plates and the subcuticular running suture of the neourethral repair. **H**, **I** Glanuloplasty with adequate caliber glans tip meatus epithelialized ventrally by the native UP and dorsally by DIG (white arrow) and closure of mucosal collar (black arrow) with healthy skin coverage. **J** Postoperative follow up with vertical slit-like glans tip meatus and epithelialized all around.** K**, **L** The same patients during evaluation by HOSE score with good outcome

In both groups, the procedure was performed under general anesthesia with caudal analgesia. In Group 1, TIP repair was conducted as described by Snodgrass (Fig. 1). In Group 2, the procedure was performed as displayed in Fig. 2 through the following steps:

### Preparation of a free mucosal graft and mucosal collar

A circumcising incision was designed to allow a 1 cm mucosal collar, and a free mucosal graft from the inner preputial surface was tailored to cover the raw area of the incised UP. Degloving was then performed, particularly in cases associated with chordee, penile rotation, or penoscrotal web.

### UP midline incision

The UP was deeply incised from the glans tip, extending proximally beyond the junction between the plate and the hypospadiac meatus.

### Graft fixation

The graft was spread to cover the raw area and secured to the edges of the UP.

### Glanular dissection

Glanular wings were deeply dissected to permit glanular closure without tension.

### Urethroplasty, spongioplasty, and dartos fascia covering

**Fig. 3:**
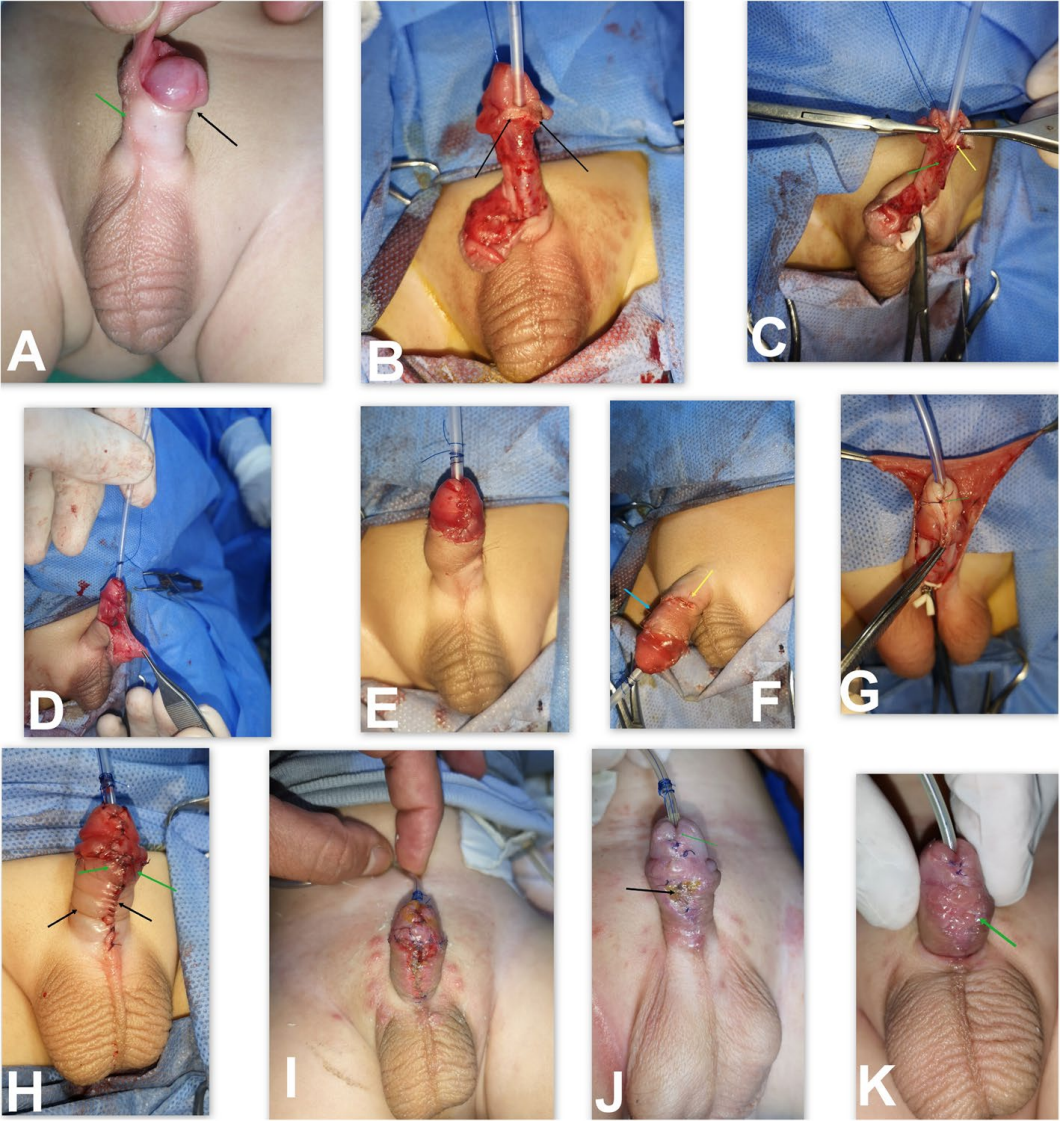
Important considerations in TIP repair. **A** Patients with coronal hypospadias associated with anticlockwise rotation (black arrow) and malformed raphe (green arrow). **B** Complete degloving with preservation of good mucosal collar and the area of modified skin around the hypospadias opening (black arrows) that after de-epithelization act as a supportive layer continuous with spongioplasty at the corona to aid in fistula prevention. **C** Starting urethroplasty proximal to the hypospadias opening at the point of Y shaped bifurcation of corpus spongiosum (green arrow), then spongioplasty done by approximation of spongiosal tissue over the urethroplasty (yellow arrow) that act as a good supportive tissue**. D** Good mucosal collar with vascularized preputial dartos flap and good vascularized skin for coverage. **E**, **F** The final picture with ventral view showing vascularized skin coverage with rotational flap and the suture line in dorsal surface after correction of penile rotation (arrows). **G** Another patient during TIP repair with ending urethroplasty at mid glans to avoid meatal stenosis. **H** The same patient at the end of surgery with tension closure of skin in the midline (arrows). **I** Appearance of devitalized skin. **J**, **K** appearance of infected granulation tissue complicated by UCF (arrow)

Urethroplasty was performed using 7/0 polyglactin subcuticular continuous sutures over a 6–8 Fr Nelaton catheter, adjusted according to patient age and glans size to avoid a tight repair in small glans. However, the use of a small-caliber catheter in a normal-sized glans may predispose to frequent obstruction by urine debris or casts. An adequate meatus was left around the urethral catheter at the glans tip, followed by spongioplasty that started at Y shaped bifurcation of corpus spongiosum through its approximation over the urethroplasty that act as a good supportive tissue with a second-layer preputial dartos fascia coverage (Fig. 3C, D).

### Glanuloplasty, mucosal collar approximation, and skin coverage

Glanular closure was initiated with deep but superficial stitches using 7/0 or 6/0 polyglactin sutures, followed by mucosal collar closure. Skin closure was performed by preparing viable skin while avoiding midline closure through the use of a vascularized preputial skin flap, thereby minimizing the risk of skin coverage complications and subsequent UCF (Fig. 3).

### Outcome assessment

In both groups, surgical outcomes were primarily compared in terms of meatal position, shape and stenosis, as well as the incidence of neourethral stricture, along with other complications, such as UCF and wound complications. Secondary outcomes included cosmetic results assessed by the hypospadias objective scoring evaluation (HOSE) score and the need for a second surgery.

### Follow-up

The first visit was scheduled for the fifth postoperative day, with daily follow-up through photos and videos sent via WhatsApp. At the second-week visit, patients attended for catheter removal after complete healing had occurred. Calibration was performed one week after catheter removal and then monthly for the first six months, with HOSE scoring conducted after this period.

### Statistical analysis

Data were collected and tabulated, and analysis was performed using SPSS version 24 (IBM, Armonk, NY, United States). Qualitative data were expressed as frequencies and percentages, while quantitative data were expressed as means ± standard deviations (SD), medians, and interquartile ranges (IQR). A p-value less than 0.05 was considered statistically significant.

## Results

### Study population and characterization

**Fig. 4:**
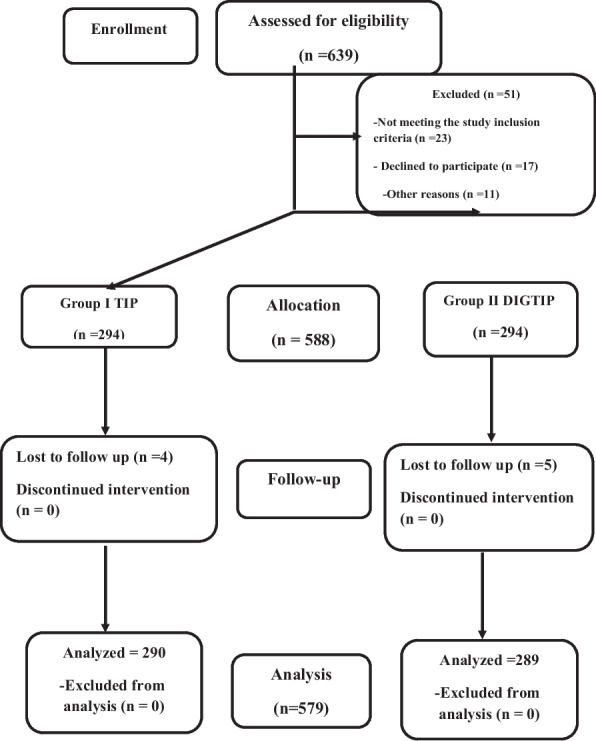
CONSORT Flow diagram

Among 639 patients with primary hypospadias without chordee who presented to the centers during the study period, 579 met the inclusion criteria (290 in Group 1 and 289 in Group 2) (Fig. 4, Flow chart). Regarding age, UP characteristics, and glans size, no statistically significant differences were observed between the two groups. The preoperative distribution of UP width in both groups was 4–8 mm in 416 patients (71.85%) and > 8 mm in 163 patients (28.15%). Patients' age, hypospadiac meatus sites, UP characteristics regarding width and extension to the glans tip, and other associated anomalies are presented in Table 1.

**Table 1:** Patients’ presentation and urethral plate (UP) characterization in both groups

Variables		Group 1 (TIP)	Group 2 (DIGTIP)	Test	*P* value
Number of patients		290	289		
1- Age (months)	Range	6–63	6–66	Z: 0.637	0.581
	Median (IQR)	22 (12.5–39)	20 (13–35)		
2- Site of hypospadic meatus:				X^2^: 7.789	0.051
- coronal		44 (15.17%)	64 (22.14%)		
- subcoronal		92 (31.72%)	88 (30.44%)		
- distal penile		144 (49.65%)	120 (41.52%)		
- mid penile		10 (3.44%)	17 (5.88%)		
3- Urethral plate (UP) characterization				X^2^: 2.991	0.084
-Width 4–8 mm		199 (68.62%)	217 (75.09%)		
- Width > 8 mm		91 (31.38%)	72 (24.91%)		
-UP that extend from hypospadias opening to glans tip		14 (19.2%)	15 (20.8%)	X^2^: 0.042	0.841
4- Associated penile anomalies				X^2^: 3.721	0.155
- Penile torsion		20 (6.9%)	11 (3.81%)		
- Penoscrotal web		66 (22.76%)	76 (26.3%)		
- Chordee corrected by degloving		98 (33.97%)	113 (39.1%)		
5- No associated penile anomalies		106 (36.55%)	89 (30.8%)	X^2^: 2.152	0.143

*IQR* Interquartile range, *Z* Mann Whitney U test, *X*^*2*^ Chi-Square test

### Operative outcomes

In both groups, a significant increase in UP width before and after the midline incision was observed, particularly in narrow plates. UP width (before and after incision) and glanular size are depicted in Table 2. Differences were noted between the two groups; however, they were not statistically significant in terms of UP width and glanular size.

**Table 2:** Different variables in operative and postoperative outcomes in both groups

Variables		Group 1 TIP (*N* = 290)			Group 2 DIGTIP (*N* = 289)			Test	*P*. value
1- UP width before incision in mm	Range	4	–	10	4	–	11	t: 0.521	0.601
	Mean ± SD	7.18	±	1.71	7.10	±	1.96		
2- UP width after incision in mm	Range	10	–	15	10	–	16	t: 1.392	0.164
	Mean ± SD	12.67	±	1.08	12.82	±	1.48		
3- Glans size mm	Range	12	–	22	12	–	21	t: 0.062	0.956
	Mean ± SD	15.62	±	1.93	15.63	±	2.37		
4- Operative time	Range	80	–	110	120	–	140	t: 64.608	0.001*
	Mean ± SD	94.05	±	6.99	129.81	±	6.31		
5- Catheter removal	Range	8	–	13	8	–	14	t: 1.562	0.120
	Mean ± SD	10.96	±	1.43	11.16	±	1.65		
6- Repair disruption	partial glanular disruption	22 (7.59%)			8 (2.7%)			X^2^: 6.841	0.009*
	complete disruption	7 (2.41%)			5 (1.73%)			X^2^: 0.331	0.564
7-The final position of the meatus	Glans tip	261 (90.00%)			276 (95.50%)			X^2^: 6.951	0.008*
	Glanular	22 (7.59%)			8 (2.7%)				
8- Meatal stenosis developed during follow up period		25 (8.62%)			3 (1.04%)			X^2^: 18.082	0.001*
- Early in the postoperative period (improved by regular dilatation)		14 (4.83%)			3 (1.04%)			X^2^: 7.291	0.001*
-Developed after 6 months (needed 2nd surgery for meatoplasty)		11 (3.79%)			0 (0%)			X^2^: 11.172	0.001*
9- Urethral stricture		5 (1.72%)			0 (0%)			X^2^: 5.029	0.025*
10- Urethrocutaneous fistula (UCF)		10 (3.45%)			5 (1.73%)			X^2^: 1.691	0.193
11- HOSE score after 6 months (score 14 or more)		221 out of 290 (76.21%)			268 out of 289 (92.73%)			X^2^: 30.118	0.001*
12- Follow up (months)	Range	6	–	23	6	–	23	t: 0.042	0.966
	Mean ± SD	13.77	±	5.69	13.75	±	5.70		

*X*^*2*^ Chi-Square test, *t* Student t test

^*^Statistically significant

Operative time was significantly longer in Group 2, with a mean of 94.05 ± 6.99 min in Group 1 versus 129.81 ± 6.31 min in Group 2 (*P* = 0.001).

The pre-incision width of the UP was not related to the size of the glans. Moreover, hypospadias is usually associated with anatomical defects such as a small glans and narrow UP; the only anatomical defect that could be corrected was UP width through incision and grafting with DIG. Catheters used in this study were 6 or 8 French, with the only significant factor influencing their choice being glans size.

Skin coverage by rotational flap was performed in 184 cases (63.44%) in Group 1 and in 200 cases (69.2%) in Group 2. Urethral catheter removal occurred in both groups after 10 to 14 days.

### Postoperative outcome assessment and complications(Table 2 and Figs. 5 and 6)

#### Meatal location

In Group 1, the meatus was located at the glans tip in 261 patients (90.0%). Partial glanular disruption with meatal regression occurred in 22 patients (7.59%); however, the meatus remained within the glans, and no further intervention was required. Complete dehiscence developed in 7 patients (2.41%).

In Group 2, the meatus was located at the glans tip in 276 patients (95.5%). Partial glanular disruption occurred in 8 patients (2.7%), resulting in an acceptable glanular position, while complete dehiscence developed in 5 patients (1.73%).

**Fig. 5:**
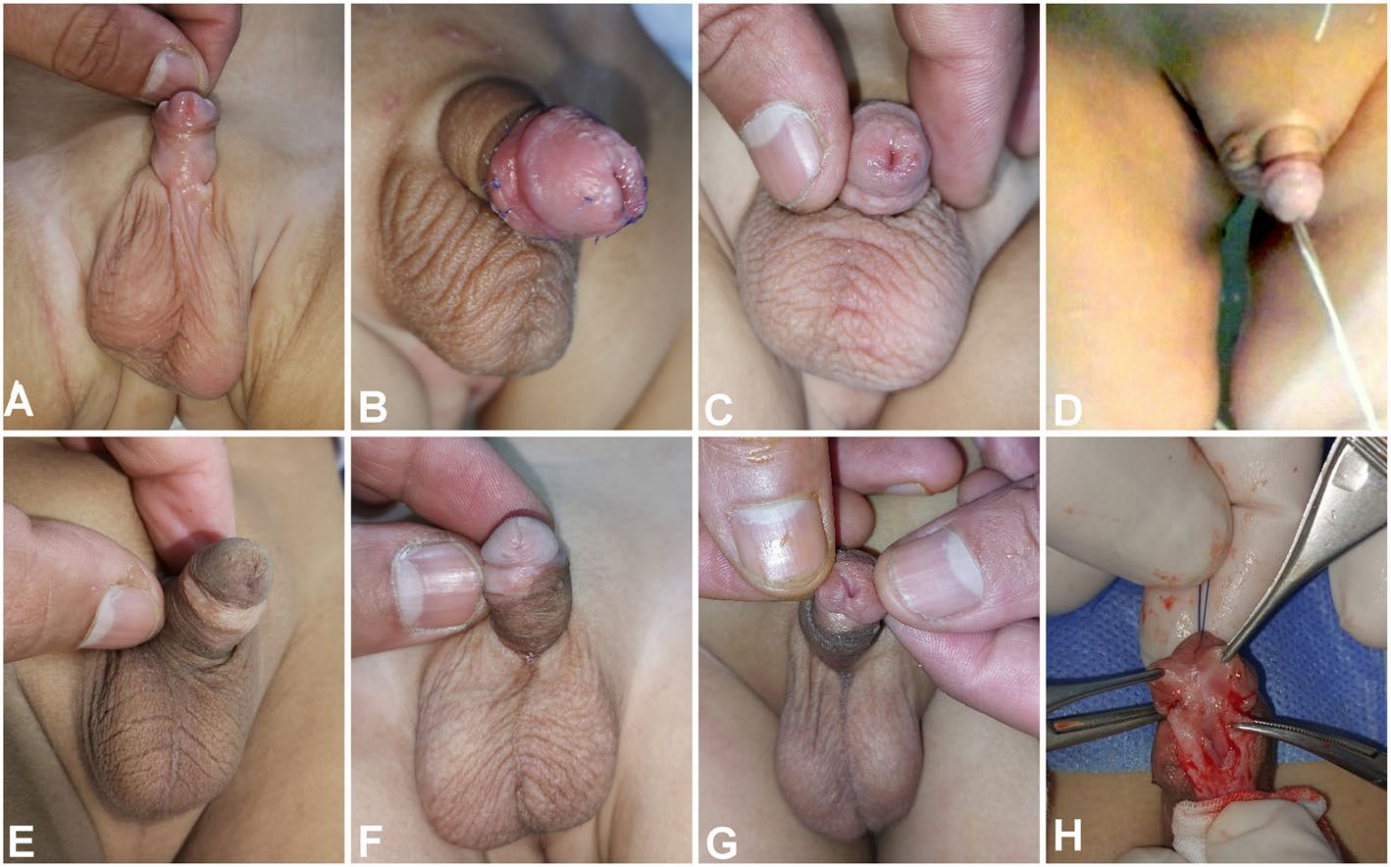
Showing the outcomes of both groups during follow up. **A** Coronal hypospadias patient with small glans and narrow UP repaired with DIGTIP. **B** Early postoperative photo of the same patient showing good outcome with vertical slit-like meatus apical glanular and conical shaped glans. **C** One year follow up of the same patient. **D** The same patient during micturition showing forward directed vertical slit-like urine stream. **E** Patient during follow up after TIP repair showing good outcome with slight meatal regression. **F**, **G** Another patient during follow up after TIP repair presented meatal stenosis that necessitate meatoplasty. **H** The same patient later developed neourethral stenosis (at coronal level) due to fibrosis and scaring with proximal urethral dilatation that necessitate redo surgery with staged inlay grafted repair

**Fig. 6:**
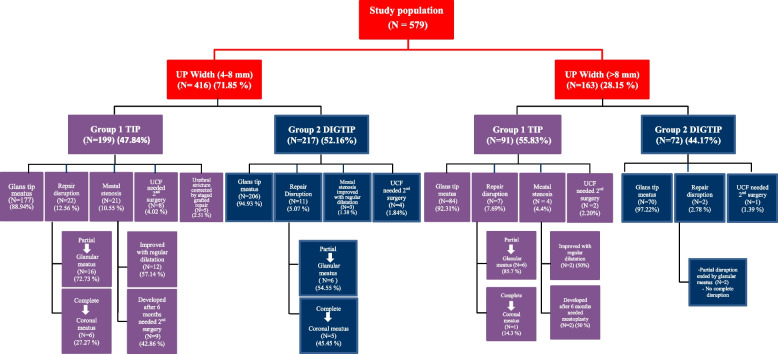
Surgical outcomes in both groups according to UP width

#### UCF

In Group 1, UCF developed in 10 patients (3.45%), whereas in Group 2, only five patients (1.73%) presented with UCF.

#### Surgical outcomes

Good surgical outcomes were defined as a vertical slit-like apical meatus with normal meatus as well as neourethral urinary function, lower complication rates, and superior cosmetic results as measured by the HOSE score. These outcomes were observed more frequently in Group 2 than in Group 1 among patients with a UP > 8 mm; however, the difference was not statistically significant. Conversely, in patients with a UP measuring 4–8 mm, Group 2 exhibited significantly better outcomes compared to Group 1, as demonstrated in Table 2 and Fig. 6. The clinical assessment included parental reports regarding the urinary stream, video recordings of micturition, and direct clinical observation. Unfortunately, uroflowmetry studies were not performed due to the young age of the study population.

#### Meatal stenosis and neourethral stricture

In Group 1, 25 patients (8.62%) developed meatal stenosis, and five patients (1.72%) developed urethral stricture, all of whom had a UP measuring 4–8 mm. In contrast, in Group 2, only three patients (1.04%) developed meatal stenosis, and no cases of neourethral stricture were reported. Among patients with a UP > 8 mm, outcomes were better in Group 2 compared with Group 1; however, the difference was not statistically significant regarding the development of meatal stenosis, repair disruption and UCF.

#### HOSE score assessment (Table 3)

**Table 3:** HOSE score assessment of surgical outcomes in both groups

Variables	Score	Group 1 TIP (*N* = 290)	Group 2 DIGTIP (*N* = 289)
1-Meatal location			
Distal glanular	4	261 (90.00%)	276 (95.50%)
Proximal glanular	3	22 (7.59%)	8 (2.7%)
Coronal	2	7 (2.41%)	5 (1.73%)
Penile shaft	1	-	-
2-Meatal shape			
-Vertical slit	2	221(76.21%)	268 (92.73%)
-Circular	1	69	21
3-Urinarystream			
-Single stream	2	221(76.21%)	268 (92.73%)
-Sprayed	1	69 (23.29%)	21 (7.27%)
4-Erection			
-Straight	4	290 (100%)	289 (100%)
-Mild angulation	3	-	-
-Moderate angulation	2	-	-
-Sever angulation	1	-	-
5-Fistula			
-None	4	273	279
-Single distal	3	10	5
-Single proximal	2	-	-
-Multiple or complex	1	-	-

The HOSE score was applied at the 6-month follow-up to evaluate both groups. This score ranges from 0 to 16 and assesses the meatal location and shape, urinary stream, straightness of erection, and presence of fistula complications.

In Group 1 (TIP), 221 of 290 patients (76.21%) achieved a score of 14 or higher. In contrast, in Group 2 (DIGTIP), 268 of 289 patients (92.73%) achieved a score of 14 or higher, representing a statistically significant improvement compared with Group 1 (*P* = 0.001).

### Follow-up and management of complications

The follow-up period ranged from 6 to 23 months (mean ± SD: 13.76 ± 5.68). In Group 1, 25 patients (8.62%) developed meatal stenosis during follow-up. Fourteen of these patients presented in the early postoperative period with a weak urinary stream secondary to meatal stenosis; they improved with regular dilatation for 2 weeks. The remaining 11 patients developed meatal stenosis after 6 months and required meatoplasty.

Another five patients (1.72%) in Group 1 developed a urethral stricture that did not respond to regular meatal dilatation or urethral dilatation under general anesthesia. These cases required surgical intervention with staged graft repair, as shown in Fig. 6. In Group 2, the three patients who developed meatal stenosis responded successfully to regular meatal dilatation.

## Discussion

DIG is an important modification adjunct to TIP repair, as it potentially prevents or minimizes stricture formation in the neourethra and/or urethral meatus. Kolon and Gonzales first described this technique in 2000, performing it on 32 patients with different types of hypospadias using a graft harvested from the inner prepuce. The neourethral tube was created in the Thiersch-Duplay fashion, and no patient developed neourethral stricture after 21 months of follow-up [11]. Grafting the incised plate with TIP repair has become an area of considerable interest. However, most studies have focused only on patients with a narrow plate, with few addressing patients with a UP wider than 8 mm. The present study aimed to determine whether DIG with TIP urethroplasty is superior to TIP alone across different types of UP [13–21].

Two studies have specifically evaluated the use of grafting the UP as an adjunct to TIP repair. Shimotakahara et al. compared DIGTIP with standard TIP repair and recommended dorsal inlay grafting as a routine step in TIP procedures [18]. Similarly, Silay et al. reported no incidence of meatal or neourethral stenosis in their cohort treated with grafted incised UP combined with TIP repair [19].

Currently, three randomized controlled trials have evaluated the outcomes of incorporating DIG into TIP repair [13, 14, 16]. The first study, conducted by Mouravas et al., included 50 patients and compared the outcomes between the grafted and non-grafted groups. They reported superior outcomes in the grafted group, with distal stenosis occurring only in TIP cases that subsequently developed fistulas [13]. In the present study, three cases (1.03%) in the grafted group developed meatal stenosis.

Conversely, Helmy et al. observed no statistically significant differences between the groups in a study involving 60 patients with UPs greater than 8 mm, except for a longer operative time in the grafted TIP group [14]. Similarly, El Deep et al., in their study of 60 patients with narrow UPs (< 8 mm), concluded that although Snodgraft was associated with longer operative time, it did not demonstrate superiority over Snodgrass. However, their analysis focused primarily on fistula incidence without reporting specific rates of meatal or neourethral stenosis or repeat surgeries [16]. In this study, meatal stenosis developed in 25 patients (8.62%) in the TIP group (Group 1) compared with three patients (1.04%) in the DIG-TIP group (Group 2), with a statistically significant difference (*P* = 0.001). Most of these cases occurred in patients with narrow UP. On the other hand, no statistically significant difference was observed in the development of meatal or neourethral stenosis between the two groups during the follow-up period, consistent with the findings of Helmy et al. [14].

Seleim et al., in their prospective study on grafting narrow plates (< 8 mm), divided patients into two groups (< 4 mm and 4–8 mm) and concluded that better results were achieved after a mean follow-up of 28.2 months, except for two cases (2.5%) of neourethral stricture, which occurred in patients with UP between 4 and 8 mm [15]. In the current study, within Group 2 (DIG-TIP), only 3 cases (1.04%) developed meatal stenosis among patients with narrow plates. By contrast, in Group 1 (TIP), meatal stenosis developed in 25 patients (8.62%), and neourethral stricture occurred in 5 patients (1.72%) during the follow-up period.

In a prospective study by Alsaid and Ahmed on 230 cases of DIG-TIP with different types of UPs and without a comparative group, no cases of meatal or urethral stenosis were reported. They documented a success rate of 96.09%, with UCF being the most common complication (3.91%) [20].

The current research supports the concept that a deep incision of the distal end of the plate, combined with preputial grafting, secures tubularization at the distal plate, resulting in an optimal slit-like meatus at the glans tip. This is supported by Ahmed et al., who reported that in DIGTIP repair, the incision is extended beyond the distal end of the UP without subsequent development of meatal stenosis during follow-up. However, Snodgrass proposed that tubularization of the UP up to the glans tip predisposes to meatal stenosis [20]. According to the researchers' experience, performing urethroplasty up to the mid-glans point may help prevent meatal stenosis in TIP repair. Still, it carries the disadvantage of glanular disruption, resulting in the subsequent development of a low-seated glanular meatus. In contrast, grafting the incised plate allows the creation of a glans tip meatus while reducing the incidence of meatal stenosis.

An important point is that grafting the incised UP improves outcomes in patients with small glans sizes. According to this study, the use of a small-caliber Nelaton catheter and 7/0 polyglactin sutures could help overcome this challenge. This finding is consistent with the results of Seleim et al. [15]. It suggests that factors related to the UP can be corrected by grafting. In contrast, a small glans size is more challenging to address and may require meticulous surgery by an experienced surgeon [21].

The present study agrees with previous reports that grafting the narrow plate (< 8 mm) is essential to prevent urethral complications, as these patients constituted 72.09% of the cases. According to this study, grafting the incised plate was associated with a significantly longer operative time and the need for additional skills that can be avoided in patients with UP width (> 8 mm). On the other hand, it provides significant value in potentially preventing meatal and neourethral stenosis, achieving an apical meatal position when extension of the UP incision is required, and avoiding the psychological trauma of postoperative meatal dilatation if needed. Thus, DIGTIP preserves the native UP and provides healthier tissue for the neourethra, as supported by many studies [11, 13, 15, 17–22].

Additionally, as previously mentioned, the width of the UP is not always uniform along its entire length, and the UP reaches the glans tip only in a minority of cases [20]. In the author's opinion, limiting the graft to the narrow plate is a restrictive factor for achieving a satisfactory outcome after TIP repair. It should be extended as a vital step in TIP repair, particularly in selected patients (when the extension of UP not reach the glans tip and/or presence of variations in UP width in the same patient) and for surgeons who achieve suboptimal results with TIP repair.

Regarding the complication rate, apart from meatal and neourethral stenosis, no significant difference was observed between the two groups. The overall UCF rate was 2.59%, which is lower than that documented in the literature [22]. In a study by Shimitokahara et al., the DIGTIP group had three UCFs, whereas the TIP group had seven UCFs [18]. This may be attributed to the use of well-vascularized second-layer coverage and viable skin flaps, which are essential factors in preventing UCF development [23].

In the cases of the current study, UCF complications occurred early due to defective mucosal collars and skin disruption in cases with midline skin closure. Dartos fascia covering the urethroplasty and vascularized skin coverage are additional important factors that the authors recommend in this study, as supported by others [24, 25].

The researchers believe that all hypospadiologists currently consider the cosmetic outcomes of hypospadias repair, particularly in distal hypospadias [23–26]. In this study, outcomes were evaluated using HOSE. Although both groups appeared similar in all steps except urethroplasty and glanuloplasty, there were differences in glans shape and meatal position. Meatal regression occurred more frequently in the TIP group 1 than in the DIGTIP group 2. Thus, there were no significant changes except in the glans score (meatal location, meatal shape, and urinary stream). Ahmed et al. utilized the HOSE score to evaluate cosmetic outcomes in their study comparing grafted TIP with classic TIP in distal hypospadias. The mean total HOSE score was 15.4 and 15.6 in grafted TIP and classic TIP, respectively [26]. In the current study, in Group 1 (TIP), 221 out of 290 patients (76.21%) achieved a score of 14 or more, while in Group 2 (DIGTIP), 268 out of 289 patients (92.73%) achieved a score of 14 or more.

### Study limitations

This study was conducted in both the short-term and mid-term phases; however, long-term results are still required and will be planned for future investigations in collaboration with additional centers. Objective evaluation of urinary function using uroflowmetry was not possible in this study due to difficulties in cooperation, as most cases were below toilet-training age (median [IQR] ages were 22 months [12.5–39] and 20 months [13–35] in groups 1 and 2, respectively). Nevertheless, this study is the first to compare DIG with TIP versus TIP repair across a wide range of different UPs.

## Conclusions

Grafting the incised plate with TIP repair can overcome anatomical variations in the UP, particularly a narrow UP width (< 8 mm), and significantly prevent both meatal and neourethral strictures. Regarding functional outcomes in UP width (> 8 mm), comparable results were achieved in both groups. DIGTIP was associated with longer operative times and higher technical demands; therefore, it should be applied only in selected patients, based on the surgeon's preference.

## Data Availability

The dataset used during the present study is available from the corresponding author upon reasonable request.

## Abbreviations

DIG: Dorsal inlay graft
DIGTIP: Dorsal inlay graft with tubularized incised plate
GTIP: Grafted tubularized incised plate
TIP: Tubularized incised plate
UCF: Urethrocutaneous fistula
UP: Urethral plate
